# Use of E-Cigarettes and Associated Factors among Youth in Thailand

**DOI:** 10.31557/APJCP.2021.22.7.2199

**Published:** 2021-07

**Authors:** Roengrudee Patanavanich, Wichai Aekplakorn, Stanton A Glantz, Rasmon Kalayasiri

**Affiliations:** 1 *Department of Community Medicine, Faculty of Medicine Ramathibodi Hospital, Mahidol University, Bangkok, Thailand. *; 2 *Center for Tobacco Control Research and Education (retired), University of California San Francisco, San Francisco, US. *; 3 *Department of Psychiatry, Faculty of Medicine Chulalongkorn University, Bangkok, Thailand. *

**Keywords:** E-Cigarettes, youth, prevalence, factors, Thailand

## Abstract

**Objective:**

The study explored e-cigarette use among youth and associated factors in Thailand.

**Methods:**

This was a cross sectional study of 6,045 seventh grade students selected using a multistage design. Self-administered questionnaires relating to the socio-demographic characteristics, history of cigarette and e-cigarette uses, friends’ and family’s use of e-cigarettes, knowledge and perception of e-cigarette use, history of alcohol uses, and life assets were gathered. Multivariate logistic regression models were used to examine the variables and their association with e-cigarette use.

**Results:**

Prevalence of ever e-cigarette use was 7.2% and current e-cigarette use was 3.7%. We found that current cigarette smoking (AOR 4.28, 95% CI: 2.05-8.94), parental e-cigarette use (AOR 6.08, 95% CI: 2.81-13.17), peer e-cigarette use (AOR 3.82, 95% CI: 2.19-6.65), peer approval of smoking (AOR 1.95, 95% CI: 1.11-3.41), and unaware of e-cigarettes’ risk (AOR 5.25, 95% CI: 2.67-10.34). were significantly associated with current use of e-cigarettes. Male sex, poor academic achievement, and poor life assets (power of wisdom) were only significantly associated with ever e-cigarette use.

**Conclusion:**

Prevalence of current e-cigarette use among Thai middle school students did not change significantly since the government banned importation and sales of e-cigarettes in 2015, suggesting that the Thai ban has been a success. Factors associated with e-cigarette use among Thai youth were consistent with other countries. Ever e-cigarette use, increased, but less than in countries without a ban. To strengthen efforts to prevent youth from e-cigarette use and addiction, the government should improve law enforcement, especially against online marketing and strengthen school-based anti-smoking programs to include e-cigarette lessons, educating parents and the public about the harm of e-cigarettes, including secondhand effects on non-users.

## Introduction

Electronic cigarettes (e-cigarettes) were first marketed in China in 2004, then became available in Europe in 2006 and the US in 2007 (Henningfield and Zaatari, 2010; Riker et al., 2012). In less than ten years since e-cigarettes were introduced, e-cigarettes have become the most commonly used tobacco product among youths in many countries (Filippidis et al., 2017; Wang et al., 2018; Brown et al., 2020; Cole et al., 2021). For example, prevalence of current e-cigarette use among US middle and high school students increased from 0.6% and 1.5% in 2011 to 10.5% and 27.5% in 2019 (Centers for Disease Control and Prevention, 2013; Wang et al., 2019). The rise of e-cigarettes has raised concerns that these products could result in a new generation of nicotine addiction (McCarthy, 2015). Moreover, studies show e-cigarettes substantially increase the risk of cigarette initiation among youth who would not otherwise become smokers (Khouja et al., 2021; Pierce et al., 2021) 

Factors associated with e-cigarette awareness, perception, and use among youth have been studied almost exclusively in high-income countries (HICs) and where sales of e-cigarettes are not banned (Kong et al., 2015; Jiang et al., 2016; Park et al., 2017; Romijnders et al., 2018). A systematic review of 17 studies on youth in HICs found that flavored e-cigarettes were perceived as being less harmful, lower costs, more fashionable and enjoyable taste than combustible cigarettes (Romijnders et al., 2018). Prior research shows that youth e-cigarette use is associated with male sex (Barrington-Trimis et al., 2015; Jiang et al., 2016; Park et al., 2017; Robert Lourdes et al., 2019; Soteriades et al., 2020), residence in urban areas (Park et al., 2017), low academic achievement (Kinnunen et al., 2020), poor knowledge about the harm of smoking (Jiang et al., 2016), positive attitudes toward e-cigarettes (Barrington-Trimis et al., 2015; Bigwanto et al., 2019), history of cigarette smoking (Dautzenberg et al., 2015; Jiang et al., 2016; Park et al., 2017; Bigwanto et al., 2019; Robert Lourdes et al., 2019; Xiao et al., 2019; Kinnunen et al., 2020; Soteriades et al., 2020), and friends’(Barrington-Trimis et al., 2015; Dautzenberg et al., 2015; Park et al., 2017; Xiao et al., 2019) and family’s members’ smoking (Barrington-Trimis et al., 2015; Dautzenberg et al., 2015; Bigwanto et al., 2019; Kinnunen et al., 2020; Soteriades et al., 2020). However, there are few studies on youth e-cigarette use in low- and middle-income countries, especially in countries that e-cigarettes are banned (Thrasher et al., 2016; Zavala-Arciniega et al., 2018; Ofuchi et al., 2020). 

Thailand has banned importation and sale e-cigarettes since 2015 (Patanavanich and Glantz, 2020). A national survey of e-cigarette use among Thai middle school students conducted in 2015 and found 3.3% (4.7% of boys and 1.9% of girls) were current (past 30-day) users (Chotbenjamaporn et al., 2017). Another survey conducted in 2019 among students aged 13-18 years in Bangkok, the capital and most populous city of Thailand, found that 6.7% of the students were current e-cigarette users (Ofuchi et al., 2020). However, factors associated with use of e-cigarettes among Thai youth are less documented. This present study provides more detailed national data collected in 2019 on e-cigarette use among youth in Thailand and examines the factors associated with the use of e-cigarettes. 

## Materials and Methods

*Data source*

The study used data obtained from 2019 Thailand Parental Supply and Use of Alcohol, Cigarettes & Drugs Longitudinal Study Cohort in Secondary School Students, which is a school-based survey assessing the risk behaviors in seventh grade students (average age 13). The survey was approved by the Institutional Review Board of Faculty of Medicine, Chulalongkorn University, Thailand. The survey used multi-stage sampling; first, simple random sampling was used to select a province in each of Thailand’s five regions (north, central, northeast, south, and Bangkok). Second, secondary schools in selected provinces were stratified according to their settings (urban public school, rural public school, and private school) and simple random sampling was used to select a school for each setting. The survey was self-administered and de-identified to preserve privacy and encourage students to report the truth. Passive parental consent was used, and student participation remained voluntary even with parental consent.

A total of 22 schools were selected; of these all students in seventh grade of selected schools were invited to participate, with 6,238 students participating in the survey, yielding a response rate of 80%. After excluding the ineligible questionnaires (inattentive responding) and subjects with missing key demographic information, 6,045 participants were included in the present study. 

*Measures*

The dependent variables were current e-cigarette use and ever e-cigarette use. Current e-cigarette use was measured by the question, “During past 30 days, on how many days did you use electronic cigarettes?” Responses were classified as 0, 1-2, 3-5, 6-9, 10-19, 20-29, and 30 days. If the response was 1 day or more, the respondent was identified as a current e-cigarette user. Ever e-cigarette use was identified if the respondent answered “yes” to the question, “Have you ever tried e-cigarettes? (yes or no).” 

Independent variables included students’ age, sex, academic achievement, parental e-cigarette use, peer e-cigarette use, peer approval of e-cigarette use, unaware of e-cigarettes’ risk, current cigarette smoking, ever cigarette smoking, current alcohol drinking, and life assets. 

Academic achievement was grade point average (GPA on a 0-4 scale) from the last semester. Parental e-cigarette use was dichotomized as “yes” if the respondent reported at least one parent uses e-cigarettes. Peer e-cigarette use was dichotomized as “yes” if the respondent reported having a classmate use e-cigarettes. Peer approval of smoking was identified if the respondent answered “no” to the question, “Do any of your closet friends mind if you smoke? (no, yes, or absolutely)”. Unaware of e-cigarettes’ risk was identified if the respondent answered “no” to the question, “Is use e-cigarettes regularly harmful to your health? (no, mild, moderate, or severe)”. Another question related to unaware of e-cigarettes’ risk was that the respondent answered “agree” to the statement, “Quitting e-cigarettes is easy (agree or disagree)”. 

Current smoker was measured by the question, “During past 30 days, on how many days did you smoke?” Responses were classified as 0, 1-2, 3-5, 6-9, 10-19, 20-29, and 30 days. If the response was 1 day or more, the respondent was identified as a current smoker. Ever smoker use was identified if the respondent answered “yes” to the question, “Have you ever tried smoking? (yes or no)”. Current alcohol drinking was measured by the question, “During past 12 months, how often did you drink alcohol?” Responses were classified as none, less than once a month, once a month, 2-3 days a month, 1-2 days a week, 3-4 days a week, 5-6 days a week, and every day. If the response was at least once a month, the respondent was identified as current alcohol drinking. 

Life assets were positive youth development measures for Thai adolescents developed by the National Institute for Child and Family Development at Mahidol University and Thai Health Promotion Foundation (Tripathi, 2013). The survey includes the life assets questionnaire that consists of 48 indicators within 5 domains (power of self, power of family, power of wisdom, power of peers, and power of community) (Tripathi, 2013). Details of each indicator are described elsewhere (Tripathi, 2013; Khirirat et al., 2020). Scores for each indicator ranged from “0” (none) to “3” (regularly).(Khirirat et al., 2020) We dichotomized the total scores into “pass” and “fail” with scores lower than 60% of the highest possible score for each domain were considered “fail” (Khirirat et al., 2020).

*Statistical analyses*

We computed weighted descriptive statistics of the characteristics of current e-cigarette users and ever e-cigarette using inverse probability weighting to provide national estimates for youth of the same age (Haugen et al., 2020). To explore the association between each dependent variable and independent variables, we conducted univariate and multivariable logistic regressions. When 95% confidence intervals (CIs) did not contain the value 1 the results were considered statistically significant (Tan and Tan, 2010). The variance inflation factor (VIF) was used to check multicollinearity (overall VIF = 1.43). Stata version 14 was used for data analysis (StataCorp, 2015).

## Results

Of the 6,045 students, 2,745 (45.4%) were boys and 3,300 (54.6%) were girls. The mean age of students was 12.9 years old (range 11-16 years); 60.0% of students were 13 years old, 27.6% were 11-12 years old, and 12.4% were 14-16 years old. In addition, 22.1% of students were from Bangkok, 25.4% were from southern region, 23.7% were from central region, 19.2% were from northern region, and 9.2% were from northeastern region. Details are reported in Table 1.

*Ever e-cigarette use*

The overall weighted prevalence of ever e-cigarette use was 7.2% (95% CI: 6.8%-7.6%). The prevalence of ever-e-cigarette use among boys was higher than girls (11.2% (95% CI: 10.7%-11.7%) vs. 2.8% (95% CI: 2.6%-3.1%)). Moreover, the prevalence of ever e-cigarette among students from central region was the highest (13.5% (95% CI: 13.2%-13.8%)) compared with other regions. Students with GPA below 3.0 were more likely to report ever e-cigarette use than students with higher GPA (15.5% (95% CI:14.7%-16.3%) vs. 4.9% (95% CI: 4.5%-5.2%)). Details are reported in Table 2. 

*Current e-cigarette use*

The overall prevalence of current e-cigarette use was 3.7% (95% CI: 3.4%-4.0%); among boys was 5.9% (95% CI: 5.5%-6.3%) and among girls was 1.3% (95% CI: 1.1%-1.4%). There was a progressive increase of current e-cigarette use with age, with the highest prevalence among 14- to 16-year-olds (6.4%, 95% CI: 5.5%-7.2%). The prevalence of current e-cigarette use was the highest among students from the central region (7.9%, 95% CI: 7.7%-8.2%). Also, 50.4% (95% CI:49.7%-51.5%) of current e-cigarette users were dual users (i.e., using both e-cigarettes and cigarettes). In addition, students with poorer GPA were more likely to be current users of e-cigarettes (Table 2). 

*Factors associated with e-cigarette use*

The multivariable logistic regression revealed that ever e-cigarette use was significantly associated with male sex (AOR 1.81, 95% CI: 1.23-2.67), poorer GPA (AOR 0.67, 95% CI: 0.48-0.93), ever cigarette smoking (AOR 10.48, 95% CI: 6.68-16.44), current cigarette smoking (AOR 4.41, 95% CI: 2.27-8.55), parental e-cigarette use (AOR 3.57, 95% CI: 1.96-6.47), peer e-cigarette use (AOR 4.26, 95% CI: 2.84-6.40), peer approval of smoking (AOR 1.93, 95% CI: 1.31-2.83), unaware of e-cigarettes’ risk (AOR 3.51, 95% CI: 1.92-6.41), and poor score on the life asset questionnaire about the power of wisdom (AOR 1.85, 95% CI: 1.12-3.06).

Current e-cigarette use among youth was significantly associated with ever cigarette smoking (AOR 4.85, 95% CI: 2.48-9.51), current cigarette smoking (AOR 4.28, 95% CI: 2.05-8.94), parental e-cigarette use (AOR 6.08, 95% CI: 2.81-13.17), peer e-cigarette use (AOR 3.82, 95% CI: 2.19-6.65), peer approval of smoking (AOR 1.95, 95% CI: 1.11-3.41), and unaware of e-cigarettes’ risk (AOR 5.25, 95% CI: 2.67-10.34). School location did not show any significant association with ever and current e-cigarette use and poor life asset scores were not significantly associated with current e-cigarette use (Table 3).

**Table 1:** Characteristics of Study Participants (Unweighted)

Characteristics	All (n=6,045)		Ever e-cigarette users (n=331; 5.8%)		Current e-cigarette users (n=115; 2.8%)	
Sex	number	%	number	%	number	%
Male	2745/6045	45.4	219/331	66.2	104/115	90.4
Female	3300/6045	54.6	112/331	33.8	51/115	44.3
Age (Mean, years)		12.9		13.0		13.0
11-12 yr old	1667/6045	27.6	64/331	19.3	31/115	20.0
13 yr old	3629/6045	60.0	216/331	65.0	96/115	61.9
14-16 yr old	749/6045	12.4	52/331	15.7	28/115	18.1
Grade point average (Mean)		3.4		2.9		2.9
Region						
Bangkok	1333/6045	22.1	75/331	22.7	31/155	20.0
Central	1431/6045	23.7	145/331	43.8	81/155	52.3
North	1159/6045	19.2	33/331	10.0	14/155	9.0
Northeast	587/6045	9.2	21/331	6.3	8/155	5.2
South	1535/6045	25.4	57/331	17.2	21/155	13.6
Cigarette						
Ever-use	406/5813	7.0	189/327	57.8	93/152	61.2
Current-use	135/5768	2.3	101/326	31	65/150	43.3
Current alcohol drinking	230/6045	3.8	80/331	24.2	47/155	30.3
Parental e-cigarette use	201/5418	3.7	51/299	17.1	34/133	25.6
Peer e-cigarette use	571/5591	10.2	154/327	47.1	86/153	56.2
Peer approval of smoking	2055/5771	35.6	223/319	69.9	106/147	72.1
Unaware of e-cigarettes' risk	186/5249	3.5	61/320	19.1	42/149	28.2
Belief quitting e-cigarettes was easy	3456/5093	67.9	242/318	76.1	111/152	73.0
Life assets (Failed)						
Power of self	1822/5519	33.0	179/302	59.3	95/141	67.4
Power of family	1535/5527	27.8	170/306	55.6	89/141	63.1
Power of wisdom	2152/5480	39.3	204/304	67.1	103/141	73.0
Power of peers	2246/5470	41.1	183/303	60.4	89/141	63.1
Power of community	3227/5466	59.0	219/303	72.3	100/141	70.9

**Table 2:** Prevalence of the Use of E-Cigarettes among Thai Grade 7^th^ Students (Weighted)

Characteristics	Ever-use				Current-use			
	Prevalence	95% CI		Weighted Number	Prevalence	95% CI		Weighted Number
Overall	7.20%	6.80%	7.60%	55,870	3.70%	3.40%	4.00%	28,034
Sex								
Male	11.20%	10.70%	11.60%	45,410	5.90%	5.50%	6.30%	23,450
Female	2.80%	2.60%	3.10%	10,460	1.30%	1.10%	1.40%	4,584
Age								
11-12 yr old	4.60%	3.70%	5.30%	7,689	2.70%	2.00%	3.30%	4,425
13 yr old	7.60%	7.10%	8.00%	38,653	3.50%	3.20%	3.90%	17,621
14-16 yr old	9.80%	8.80%	10.70%	9,528	6.40%	5.50%	7.20%	5,989
Grade point average								
Below 3.0	15.50%	14.60%	16.30%	26,358	8.40%	7.60%	9.00%	13,875
3.0 – 4.0	4.90%	4.50%	5.20%	29,513	2.40%	2.10%	2.70%	14,160
Region								
Bangkok	6.20%	6.00%	6.30%	3,388	2.60%	2.50%	2.70%	1,397
Central	13.50%	13.20%	13.80%	34,222	7.90%	7.70%	8.20%	19,490
North	3.10%	3.00%	3.10%	3,980	1.40%	1.40%	1.40%	1,736
Northeast	4.30%	4.10%	4.50%	9,316	1.70%	1.60%	1.80%	3,624
South	4.10%	4.00%	4.10%	4,965	1.50%	1.50%	1.50%	1,787
Cigarette								
Ever-use	49.90%	49.70%	50.10%	32,075	27.00%	26.20%	27.70%	16,913
Current-use	73.60%	71.20%	77.00%	17,011	50.40%	49.70%	51.50%	11,511

**Table 3:** Factors Associated with Ever and Current E-Cigarette Use

Independent variables	Ever e-cigarette								Current e-cigarette							
	Unadjusted OR	[95%CI]		p-value	AOR*	[95%CI]		p-value	Unadjusted OR	[95%CI]		p-value	AOR*	[95%CI]		p-value
Sex																
Male	2.58	2.04	3.26	< 0.001	1.81	1.23	2.67	0.003	2.63	1.87	3.69	< 0.001	1.64	0.94	2.86	0.084
Female	1.00								1.00							
Age (years)	1.38	1.16	1.65	< 0.001	1.15	0.83	1.58	0.405	1.42	1.11	1.83	0.006	1.14	0.70	1.87	0.594
Grade point average	0.29	0.24	0.35	< 0.001	0.67	0.48	0.93	0.017	0.30	0.24	0.39	< 0.001	0.84	0.53	1.32	0.437
Region																
Bangkok	1.55	1.09	2.21	0.015	1.1	0.62	1.95	0.751	1.75	1.00	3.06	0.051	1.22	0.48	3.07	0.678
Central	2.92	2.13	4.01	< 0.001	1.63	0.94	2.82	0.08	4.41	2.71	7.18	< 0.001	2.31	0.97	5.50	0.059
North	0.74	0.48	1.15	0.182	0.54	0.26	1.14	0.108	0.89	0.45	1.75	0.726	0.69	0.21	2.24	0.534
Northeast	0.90	0.54	1.50	0.691	0.59	0.25	1.42	0.238	0.94	0.41	2.14	0.884	1.12	0.34	3.71	0.858
South	1.00								1.00							
Cigarette																
Ever-use	34.78	26.80	45.15	< 0.001	10.48	6.68	16.44	< 0.001	27.42	19.38	38.79	< 0.001	4.85	2.48	9.50	< 0.001
Current-use	75.96	49.71	116.07	< 0.001	4.41	2.27	8.55	< 0.001	61.05	40.76	91.44	< 0.001	4.28	2.05	8.94	< 0.001
Current alcohol drinking	13.74	10.08	18.72	< 0.001	1.9	1	3.63	0.05	14.87	10.19	21.71	< 0.001	1.70	0.8	3.63	0.171
Parental e-cigarette use	6.99	4.96	9.85	< 0.001	3.57	1.96	6.47	< 0.001	10.61	6.97	16.14	< 0.001	6.08	2.81	13.17	< 0.001
Peer e-cigarette use	10.44	8.22	13.26	< 0.001	4.26	2.84	6.4	< 0.001	13.56	9.72	18.93	< 0.001	3.82	2.19	6.65	< 0.001
Peer approval of smoking	4.63	3.62	5.92	< 0.001	1.93	1.31	2.83	0.001	4.93	3.42	7.10	< 0.001	1.95	1.11	3.41	0.019
Unaware of e-cigarettes' risk	9.08	6.52	12.64	< 0.001	3.5	1.92	6.41	< 0.001	13.52	9.12	20.06	< 0.001	5.25	2.67	10.34	< 0.001
Belief quitting e-cigarettes was easy	1.54	1.18	2.01	0.001	1.49	0.98	2.26	0.065	1.29	0.9	1.85	0.171	1.11	0.60	2.05	0.744
Life assets (Failed)																
Power of self	3.19	2.52	4.05	< 0.001	0.75	0.45	1.26	0.283	4.37	3.06	6.25	< 0.001	1.14	0.58	2.23	0.71
Power of family	3.58	2.83	4.52	< 0.001	1.36	0.85	2.18	0.198	4.71	3.33	6.67	< 0.001	1.40	0.71	2.76	0.335
Power of wisdom	3.42	2.68	4.38	< 0.001	1.85	1.12	3.06	0.017	4.37	3.00	6.36	< 0.001	1.67	0.82	3.40	0.156
Power of peers	2.31	1.83	2.93	< 0.001	0.77	0.46	1.31	0.341	2.53	1.79	3.57	< 0.001	0.82	0.39	1.73	0.605
Power of community	1.88	1.45	2.43	< 0.001	0.86	0.51	1.44	0.564	1.71	1.18	2.47	0.004	0.54	0.25	1.17	0.118

Adjusted for all variables shown in the Table.

## Discussion

Thailand has banned import and sales of e-cigarettes since 2015, mainly to protect youth from addiction (Patanavanich and Glantz, 2020). The continued low prevalence of current e-cigarette use among youth we found in this study could be the effect of the ban because use was not statistically significantly different from the prevalence from the 2015 survey (3.7% in 2019 vs. 3.3% in 2015 (Chotbenjamaporn et al., 2017), p=0.359. This finding is consistent with previous studies indicating that the change in prevalence of e-cigarette use among countries where the products were banned was slower than the countries where the products were allowed (Gravely et al., 2014; Gravely et al., 2019). For comparison, in the US, where e-cigarettes were legal, the prevalence of current e-cigarette use among middle school students nearly tripled from 3.9% in 2014 (Arrazola et al., 2015) to 10.5% in 2019 (Cullen et al., 2019). However, it is noteworthy that current e-cigarette use among Thai youth was relatively high compared with other countries where e-cigarettes are also banned (e.g. Brazil: 0.7% (Bertoni et al., 2019); Australia: 1.8% (Greenhalgh et al., 2020)). Furthermore, the significant increase of ever e-cigarette use from 5.4% in 2015 (Chotbenjamaporn et al., 2017) to 7.2% in 2019 (p=0.001), while smaller than in the US, is concerning. 

One explanation for the higher prevalence of e-cigarette use in Thailand compared to other countries that ban e-cigarettes could be low compliance with the ban on tobacco (including e-cigarettes) advertising, promotion, and sponsorship (TAPS) in Thailand, especially via online social media platforms, the most common channel the tobacco industry uses to target youth (Ketonen and Malik, 2020; Vogel et al., 2021). According to the 2019 World Health Organization’s report on the global tobacco epidemic, Thailand’s compliance with bans of TAPS was 6/10 (whereas Brazil 9/10 and Australia 10/10) (World Health Organization, 2019). The government must continuously monitor the promotion of e-cigarettes and strengthen enforcement of TAPS, especially online platforms to prevent youth from initiating it. 

Consistent with prior studies in other countries (Barrington-Trimis et al., 2015; Dautzenberg et al., 2015; Jiang et al., 2016; Park et al., 2017; Bigwanto et al., 2019; Robert Lourdes et al., 2019; Xiao et al., 2019; Kinnunen et al., 2020; Soteriades et al., 2020), we found male sex, poor academic achievement, cigarette smoking, parental use of e-cigarettes, peer use of e-cigarettes, peer approval of smoking, and unaware of the risk of e-cigarettes were independently associated with the use of e-cigarettes among Thai youth. These findings are important and could be used for planning effective e-cigarette prevention programs for youth. 

The association of parental e-cigarette use and an increased risk of e-cigarette use in offspring is similar to cigarette smoking (Vuolo and Staff, 2013). Children of smoking parents are more likely to initiate cigarette smoking (Vuolo and Staff, 2013; Wu and Chaffee, 2020). However, parents are less aware when their children use e-cigarettes than when they smoke (Wu and Chaffee, 2020). and those who use e-cigarettes may perceive that e-cigarette aerosol is safer for their children (Drehmer et al., 2019). Studies show that secondhand exposures to e-cigarette aerosol result in significant changes in biomarker concentrations such as nicotine (Johnson et al., 2019; Son et al., 2020; Quintana et al., 2021), acrolein (Johnson et al., 2019), tobacco-specific nitrosamines (Quintana et al., 2021) and airborne PM_2.5_ (Son et al., 2020). Health risks of e-cigarettes to bystanders include upper respiratory tracts and eyes’ irritation and systemic effects of nicotine such as increased heart rate and blood pressure (Visser et al., 2019). Public education programs and tobacco control advocates could use this finding to inform parents that using e-cigarettes is not safe for children and likely to increase children’s initiation of e-cigarettes. This information could also be used in public education programs to empower the non-using majority of youth to object to others’ using e-cigarettes in their presence, which would reduce the social acceptability of e-cigarette use.

In addition to parental influence on youth use of e-cigarettes, peers’ normative influence toward e-cigarette use was consistent with smoking that peers’ descriptive (peers smoke) and injunctive norms (peers approve of smoking) contribute to adolescent smoking (Scalici and Schulz, 2017). School-based social competence (improving students’ social competence and social skills that help students refuse offers to smoke) and social influence curricula (teaching students to be aware of social influences and dealing with peer pressure that encourage smoking) have been effective in preventing the initiation of youth smoking (Thomas et al., 2015). In addition, programs to educate youth about secondhand effects of e-cigarette use on non-users can mobilize peer pressure supporting non-use. These approaches should be applied to e-cigarette use. Other innovative approaches such as using social or friendship networks and informal peer vaping prevention messages also show promising outcomes such as the peer-led Above the Influence of Vaping (ATI-V) in the U.S. (Wyman et al., 2021).

Poor knowledge of the harm of e-cigarettes is also concerning. This study emphasizes that students who are unaware of the risk of e-cigarettes are more likely to be e-cigarette users. Prior studies also note that young people who use e-cigarettes are less likely to report that e-cigarettes are harmful to their health (Gorukanti et al., 2017; Bernat et al., 2018). Public education and school programs could be a good place to educate students about the harm of e-cigarettes as 76.2% of Thai students aged 13-15 years report that they learn about dangers of tobacco use in school (Chotbenjamaporn et al., 2017). Furthermore, we found that students with poorer scores on the power of wisdom (schools’ support, safety, clear rules, and students’ attitudes toward learning and schools’ loyalty) (Tripathi, 2013; Khirirat et al., 2020) were more likely to use e-cigarettes. It is also concerning that over half of students were dual users of cigarettes and e-cigarettes because dual use is more dangerous to respiratory and cardiovascular systems than smoking or e-cigarette use alone (Alzahrani et al., 2018; Osei et al., 2019; Wills et al., 2021). 

In Thailand, the school-based anti-smoking education programs or the smoke-free schools focus on cigarette smoking and information about the risk of e-cigarette in Thai language is limited. E-cigarette prevention programs in schools such as the CATCH My Breath program in the U.S. is found to be effective in lower prevalence of e-cigarette users (Kelder et al., 2020). Moreover, public education messages on e-cigarettes are also needed. For example, the Real Cost youth e-cigarette prevention media campaign of the U.S. Food and Drug Administration is a good example that reduces intentions and attitudes toward e-cigarette using and increases perception of the risk of e-cigarettes among young people among at-risk youth who have already experienced smoking and those who have not yet initiated smoking (Noar et al., 2020). There is also a need to reduce social acceptability of e-cigarette use by educating people about the effects of secondhand exposure on non-users. Furthermore, e-cigarette cessation and treatment programs are also needed, but not yet widely available even in the developed countries (Gaiha and Halpern-Felsher, 2021). To our knowledge, there are no specialized cessation programs for youth use of e-cigarettes in Thailand.

*Limitations*

We were unable to identify causality of e-cigarette use and relevant factors due to the cross-sectional study design. The questionnaire is lengthy and based on self-reported data; thus, could be subject to reporting errors. This study focused on seventh grade students, future studies could extend the survey to older students as reported in other countries that e-cigarettes are more popular among high school compared with middle school students. However, the present study has its strength as including a large representative sample size for secondary school children in Thailand.

In conclusion, prevalence of current e-cigarette use among Thai middle school students remained low since the government banned e-cigarette import and sale in 2015. Ever e-cigarette use increased, albeit less than in countries without bans. To reinforce the effectiveness of the ban on imports and sales to prevent youth from e-cigarette use, a comprehensive approach is required. Improving law enforcement against online advertising and marketing on e-cigarettes is an important task for the government. In addition, strengthening school-based anti-smoking programs to include e-cigarette lessons, implementing social networks by peer-led e-cigarette prevention messaging campaigns, developing mass media campaigns to educate parents and the public about the harms of e-cigarettes, including secondhand exposure to non-users, are essential to protect young generations from e-cigarette use and addiction. In addition, e-cigarette cessation and treatment programs for youth should be prepared and implemented.

## Author Contribution Statement

RP developed the idea for the study, analyzed the data, and wrote the first draft of the manuscript. SAG and WA assisted with revising and refining the manuscript. RK provided data and assisted with revising the manuscript.

## Data Availability

All data used to prepare this paper are available from the cited sources. The 2019 Thailand Parental Supply and Use of Alcohol, Cigarettes & Drugs Longitudinal Study dataset can be requested directly from the Thailand Parental Supply and Use of Alcohol, Cigarettes & Drugs Longitudinal Study Cohort in Secondary School Students’ Project, Department of Psychiatry, Faculty of Medicine Chulalongkorn University, Bangkok, Thailand.
